# How does the brain age in individuals with multiple sclerosis? A systematic review

**DOI:** 10.3389/fneur.2023.1207626

**Published:** 2023-06-30

**Authors:** Nataliya Tokarska, Isabelle Tottenham, Charbel Baaklini, Jodie R. Gawryluk

**Affiliations:** ^1^Department of Anatomy, Physiology and Pharmacology, University of Saskatchewan, Saskatoon, SK, Canada; ^2^Department of Clinical Neurosciences, University of Calgary, Calgary, AB, Canada; ^3^Department of Neuroscience, University of Alberta, Edmonton, AB, Canada; ^4^Department of Psychology, University of Victoria, Victoria, BC, Canada

**Keywords:** multiple sclerosis, aging, magnetic resonance imaging, systematic review, brain

## Abstract

Multiple Sclerosis (MS) is a complex neurological disorder that involves demyelination, lesions and atrophy in both white and gray matter. Such changes in the central nervous system are diagnostic in MS and has a strong relationship with both physical and cognitive symptoms. As a result, magnetic resonance imaging (MRI) scans as a metric of brain atrophy have emerged as an important outcome measure in MS studies. Recently, research has begun to focus on the contribution of aging to the structural changes in the brain associated with MS; prompting questions about whether there is an amplifying effect of aging superimposed on MS-related brain atrophy. To examine current evidence of how the brain ages in individuals with MS, a systematic review of the literature was performed. Specific questions were focused on how aging affects gray and white matter structure, whether patterns of brain atrophy differ in younger and older cohorts and if there are structural differences in the brain as a function of sex in aging people with MS. This review considered studies that used MRI to examine the effects of aging in adults with MS. Twenty-one studies met eligibility criteria. Findings across these studies revealed that gray matter atrophy was more pronounced in older adults with MS, particularly in subcortical regions such as the thalamus; that the rates of atrophy were similar but varied by region for younger and older cohorts; and that males may experience more brain atrophy than females. Further studies that use multimodal MRI acquisition methods are needed to capture changes in both males and females over time, particularly in middle to older adulthood.

## Introduction

Globally, the population is aging at an unprecedented rate (1). Given the shifts in population demographics, it is essential to better understand the neurobiological aging process in both healthy conditions and in people with neurological disorders. Recent research has used magnetic resonance imaging (MRI), a non-invasive and easily repeatable technique, to depict normative trajectories of aging in gray and white matter. Specifically, Bethlehem et al. (2) used MRI data from more than 100,000 brain scans to create brain charts revealing patterns of gray and white matter atrophy in both males and females throughout middle and late adulthood (age 40 years onwards). These findings were largely in line with previous research indicating that after the age of 40 years, overall brain volume begins to decline by a rate of 5% per decade, with increased rates of decline at 70 years onwards (3–6). These are expected changes in the brain with normative aging. Although a better understanding of the neurobiological underpinnings of aging is being developed, relatively less is known about how the brain changes with age in people with chronic neurological conditions, such as multiple sclerosis.

Multiple Sclerosis (MS) is an inflammatory demyelinating disorder which predominantly affects the central nervous system (CNS). Approximately 90% of MS cases initially present as relapsing–remitting (RRMS), with acute inflammation and demyelinating lesions associated with relapsing symptoms (7). Over time, the majority of people with MS (pwMS) develop secondary progressive symptoms (SPMS), and although less common, some individuals present with primary progressive symptoms from onset (PPMS). Notably, MS tends to affect women more than men at an approximate 3:1 ratio, although, men who develop MS tend to move toward progressive stages of the disease faster (8). Regardless of the subtype, MS is characterized by chronic and cumulative changes in the brain that are evident on magnetic resonance imaging (MRI) and represent a key diagnostic criterion. Specifically, diagnoses are based on MRI-detected lesions (which tend to occur in periventricular regions) disseminated in time and space, in conjunction with characteristic symptoms. From a research perspective, common MRI findings for pwMS include decreased whole brain volume, enlarged ventricles, and a measurable white matter lesion load, as well as a decreased white matter integrity on diffusion tensor imaging (DTI) (9). MRI metrics have emerged as important outcome measures in MS studies, given that brain atrophy and white matter lesions are strong predictors of motor impairment and cognitive dysfunction (10). Unlike many other neurological disorders, MS is typically diagnosed in young to middle adulthood. Yet, most pwMS live into older adulthood, with life expectancy at approximately 75 years (11). Along with the population as a whole, the proportion of older adults with MS is growing and many older adults with MS did not have access to disease modifying treatments at the time of their diagnosis (12).

Age is an important factor when considering the disease course of MS, especially given that pwMS over the age of 65 are more commonly in the progressive form of the disease (13). Reduced remyelinating capacity is observed independently in aging populations and in pwMS. It has been shown in animal models of MS that as age increases, oligodendrocyte precursor cells, the primary cells responsible for remyelination, have reduced capacity for differentiation into remyelinating oligodendrocytes (14). Further, iron accumulation and deposition are hallmarks of not only normal aging, but older pwMS tend to show exacerbated iron toxicity that can cause increased neurodegeneration (15). Additionally, it has been suggested that inflammation related to previous viral infections (e.g., Epstein–Barr virus) can result in decreased immune system function and reduced capacity for tissue repair (12, 16).

Essentially, emerging research has begun to focus on the contribution of normal aging to the structural changes in the brain associated with MS; prompting questions about whether there is an amplifying effect of aging superimposed on MS-related neurodegeneration. To examine current evidence, a systematic review of the literature that used MRI to examine aging in pwMS was performed. This is the first study to synthesize the literature on the effects of aging on brain structure in pwMS. The following questions were specifically examined and will be discussed in this review:

1. How does brain structure (e.g., volume, white matter microstructure) differ or change with age in pwMS?
2. Are there specific structural MRI findings in older pwMS compared to younger pwMS?
3. Are there structural differences in the brain as a function of sex in aging pwMS?

It was hypothesized that there would be differences in brain structure that were specific to pwMS beyond the changes associated with typical aging and that older pwMS would have greater whole brain atrophy than younger pwMS. Sex differences were examined because of the known differences in prevalence and progression of MS between females and males, although this question was purely exploratory.

## Methods

### Eligibility criteria

Specific eligibility criteria included studies that were peer-reviewed, in English, focused on adult (>18 years of age) human subjects and included an MS group (any subtype: RRMS, SPMS, PPMS) with full-text availability. Given the research questions of interest, studies must have also used MRI to examine brain structure. Any acquisition sequence was acceptable (e.g., T1-weighted, T2-weighted, diffusion weighted, FLAIR) because each provides different information about the brain. For example, T1-weighted scans can be used to examine gray matter volume, and diffusion-weighted scans can be used to examine white matter microstructure. Exclusion criteria included interventional studies and any studies focused on participants with other neurodegenerative conditions with comorbid MS. This review was registered on PROSPERO (CRD42021287667).

### Search strategy

The literature search was conducted using two databases, PubMed and PsycINFO. Several preliminary search strategies were tested to determine the strategy that would be inclusive yet precisely able to identify papers of interest. The search strategy used a combination of terms relevant to the research questions and agreed upon by the authors (see Supplementary material for search terms in full). In summary, search terms included variables of interest including “aging,” “multiple sclerosis,” “white matter” “gray matter,” and “MRI.” Different variations of search terms were used to account for differing spellings and key words across articles. The reference sections of several articles were also screened to ensure that the search strategy was sufficient.

### Screening strategy

Titles and abstracts of search results were extracted and uploaded into systematic review management software, Covidence. Duplicates were removed. An initial screening of the titles and abstracts was conducted based on the eligibility criteria stated previously to exclude ineligible articles. The suitability of each article based on title and abstract was reviewed by two randomly selected authors (two of NT, IT, CB, JG) such that no more than one quarter of the articles were reviewed by the same two reviewers. Where disagreements occurred regarding eligibility of an article, a randomly selected third author would break the tie and determine the final eligibility of the article. Full-text articles were then uploaded and a second screening was conducted by two authors based on the entire research article using the same eligibility criteria. As per the initial screening, a randomly chosen third author resolved any conflicts.

### Study quality ratings

To assess the quality of the articles included in this systematic review, the well-established critical appraisal tool for cross-sectional studies (AXIS) was used to rate each study. The AXIS tool uses a set of 20 questions that evaluate study design quality and quality of reporting. Studies were randomly assigned to authors, with each study assessed by a single author (one of NT, IT, CB, JG). Each study was assessed and interpreted for each of the 20 questions to evaluate its overall quality. To maintain consistency, both cross-sectional and longitudinal studies were assessed using the AXIS tool to rate the quality.

### Data analysis and extraction

Studies were assigned to authors randomly (one of NT, IT, CB, JG) to extract relevant data. For each study, the information extracted included sample size, MS group subtypes at baseline, mean age at baseline, sex ratio, geography of study participants, study type (cross-sectional or longitudinal), acquisition/analysis measurement methods used in the study, and summarized results relevant to the research questions identified for this review.

## Results

### Systematic review

**Figure 1:**
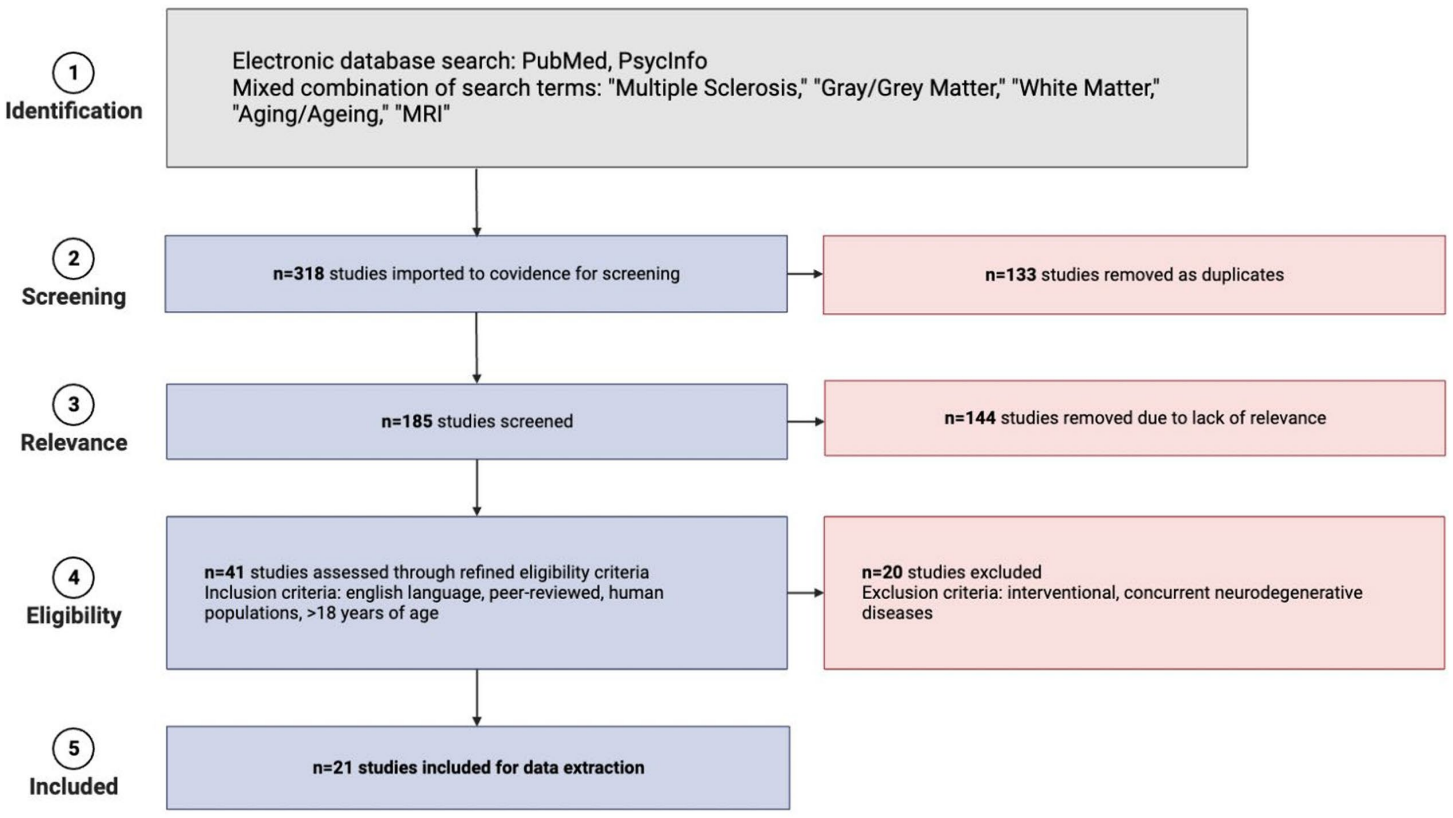
PRISMA flow diagram detailing the steps of literature collection for this systematic review (created in BioRender).

The search for studies was conducted on September 22, 2021. Initial search results yielded 318 studies. Covidence software automatically removed 133 duplicate studies based on titles. A total of 185 articles remained and their titles and abstracts were manually reviewed and screened further for inclusion and exclusion criteria, after which 144 irrelevant articles were excluded. Manual retrieval of full-text articles was conducted for the remaining 41 studies. These 41 full-text articles were then reviewed in-depth for inclusion. Each article was screened by two authors and any discrepancies for inclusion were resolved by a third author who was not involved in the initial voting for that article. Exclusions primarily resulted when studies did not have appropriate outcomes for this review (e.g., interventional studies, studies in which participants had concurrent neurodegenerative disorders, etc.). Supplementary Figure S1 details the papers that underwent full-text review but were ultimately excluded. The selection process is detailed in Figure 1 using a PRISMA flow diagram. A total of 21 studies published between 2003 and 2021 were included in the final review. Table 1 provides all the information extracted from the 21 full-text articles including sample size, MS groups, mean age, sex, geography, study type, acquisition/analysis, and summary of study results.

**Table 1:** Characteristics of papers included in this review.

Study	Sample size	MS groups (at baseline)	Mean age (years)	Sex (M:F)	Race/Ethnicity	Expanded disability status scale	Disease modifying treatments	Geography	Study type (duration)	Acquisition (resolution of T1 scan provided)	Summary of results
Azevedo et al. (17)	520 MS/CIS 81 HC	90 CIS 392 RRMS 38 SPMS	MS: 41.1 HC: 42.7	183:418	Not reported	Baseline: 1.8 (1.5)	22.3% no DMT; 77.7% up to 5 years of DMT	United States	LONG (4.1 years)	3T 3D T1-weighted 1 mm^3^ resolution	Baseline thalamic volume reduced in pwMS compared to HCs. pwMS have significantly higher rates of thalamic atrophy compared to HC aging over time. Thalamic atrophy correlates with increase in disability. DMT use at baseline did not significantly impact the rate of thalamic decline.
Azevedo et al. (18)	520 MS/CIS 130 HC	90 CIS 392 RRMS 38 SPMS	42.7	155:365	Not reported	Baseline: 1.8 (1.5)	22.3% no DMT; 77.7% up to 5 years of DMT	United States	XS LONG (5 years)	3T 3D T1-weighted 1 mm^3^ resolution	GM structures are the top ranked regions of MS atrophy, including total GM volume, thalamus, putamen and caudate. Compared to HC aging, MS atrophy contribution seems to decrease from ages 30 to 60. In the thalamus, HC normal aging contribution to atrophy increases over time while MS contribution to atrophy decreases over time. In the putamen and caudate, HC aging and MS atrophy were more stable in their contributions to structural atrophy over time.
Baird et al. (19)	31 MS 22 HC	29 RRMS 2 PMS	MS: 63 HC: 63.7	11:42	83.9% Caucasian; 16.1% African American	4.0 (1.5)	22.6% no DMT; 77.4% yes DMT	United States	XS	3T 3D T1-weighted 1 mm^3^ resolution	Mobility was significantly worse in older MS group compared to age-matched HC. There were no differences in GM volumes between MS and HC groups. Normal WM volume was significantly decreased and ventricular CSF was significantly increased in MS group, as compared to HC. In both groups, there was no correlation between brain atrophy or cognitive function with mobility.
Bishop et al. (20)	38 MS 52 HC	36 RRMS 2 SPMS	“Young” MS: 30.4 “Older” MS: 48.7 “Young” HC: 31.9 “Older” HC: 47.3	37:53	Not reported	“Young MS” 3.0 (1.2); “Older MS” 4.1 (1.3)	Not reported	United Kingdom	XS	3T 3D T1-weighted, FLAIR 1 mm^3^ resolution	Brain volume loss in all MS groups compared to HCs was predominantly in the subcortical GM. Young and older MS groups showed similar, strong excess volume loss in the putamen, thalamus and nucleus accumbens, compared to HC aging. No excess volume loss was detected in the amygdala or pallidum. The young MS group also showed significant excess volume loss in the hippocampus and caudate compared to the young HC group.
Bove et al. (21)	551 MS/CIS	481 RRMS/CIS 26 PPMS 43 SPMS	“Young”: 42 “Older”: 58	140:411	Young 95.2% white; 3.6% Hispanic Older 95.4% white; 0.7% Hispanic	Young 1.42 (1.33) Older 2.39 (1.99)	Not reported	United States	XS LONG (2 years)	1.5T 3D T2-weighted 0.93 × 0.93 × 3 mm voxels	Whole brain atrophy increases with age in MS groups. Brain atrophy and disease severity was consistently higher in men compared to women in all MS groups. There were no significant differences between the young and older MS groups and no acceleration of decline with age. No interactions were found between age and sex.
Cole et al. (22)	1,204 MS/CIS 150 HC	296 CIS 677 RRMS 111 SPMS 120 PPMS	MS: 39.41 HC: 37.29	501:853	Not reported	CIS 1.36 (1.02) RRMS 2.12 (1.40) SPMS 5.83 (1.20) PPMS 5.10 (1.32)	CIS 20% yes 79% no RRMS 53% yes 42% no SPMS 46% yes 47% no PPMS 7% yes 87% no	Netherlands, Catalonia, Switzerland, Austria, United Kingdom, Italy	LONG (HC: 1.97 years.; MS: 3.41 years) *retrospective*	1.5T and 3TT1-weighted Resolution not reported	MS group had significantly higher brain predicted age difference (brain-PAD) compared to HCs (10.3 years vs. 4.3 years). The highest brain-PADs were for people with SPMS (13.3 years). Higher brain-PAD reflect accelerated aging. Greater annual brain-PAD increases were also associated with higher disease severity.
Erramuzpe et al. (23)	10 MS 170 HC	Not stated	Not stated Age Range: 6–81 (10 HC children <18)	4:6	Not reported	Not reported	Not reported	United States	XS (HC) LONG (MS; 2 years)	3T 3D T1-weighted, FLAIR 0.93 × 0.93 × 3 mm voxels	Compared to HCs, MS group was predicted to have a higher biological age than their chronological age with the R1 (longitudinal relaxation based) age prediction method, even when only looking at normal appearing tissue. Differences between groups were revealed in parietal and occipital regions. The MS group also showed deficits in cortical thickness in midfrontal regions, compared to HC. The authors concluded that the MS group exhibited faster brain aging compared to HC aging.
Eshaghi et al. (24)	36 MS 19 HC	36 PPMS	MS: 42.8 HC: 37.6	34:21	Not reported	4 (min 1.5, max 7)	Not reported	United Kingdom	LONG (1,2,3,5 years)	1.5T 3D T1-weighted, T2-weighted 1.5 × 1.173 × 1.17 mm voxels	There was a marked progression of atrophy in several GM regions over time in the MS group compared to HC aging. Patients showed a greater decline of GM volume bilaterally in the cingulate cortex, thalamus, putamen, precentral gyrus, insula and cerebellum compared to HCs over five years. The progression of GM atrophy in the MS group occurs at different rates in different regions across the brain, with the fastest atrophy seen in the cingulate cortex and the slowest in the precentral gyrus. Some regions showed a significant volume loss at a later time point than other regions. There was a significant association between rate of volume loss in the cingulate cortex and worse clinical outcome at the five year time point.
Eshaghi et al. (25)	1,214 MS/CIS 203 HC	253 CIS 708 RRMS 128 SPMS 125 PPMS	MS/CIS: 39.2 HC: 38.7	531:886	Not reported	CIS 1 (range 0–4.5) RRMS 2 (range 0–7) SPMS 6 (range 2.5–9) PPMS 5 (range 2–8)	CIS 20% yes RRMS 49% yes SPMS 41% yes PPMS 6% yes	Netherlands, Catalonia, Switzerland, Austria, United Kingdom, Italy	LONG (HC: 1.43 years.; MS/CIS: 2.41 years) *retrospective*	3T 3D T1-weighted, FLAIR 1 mm^3^ resolution (varied slightly at different sites)	SPMS group had the lowest baseline volumes of cortical/deep GM (thalamus, putamen, globus pallidus, caudate, and amygdala) of all MS groups compared to HC. SPMS group showed a faster rate of temporal GM atrophy compared to RRMS, CIS and HC groups. SPMS group also showed faster parietal GM atrophy than the CIS group and HC. RRMS, SPMS and PPMS groups showed faster rates of deep GM atrophy and whole cortical GM compared to the CIS group and HC. However, deep GM rate of atrophy was higher than cortical/cerebellar GM and brain stem. Deep GM atrophy rate correlated with disability progression. SPMS and PPMS groups showed higher disability compared to RRMS and CIS groups.
Fisher et al. (26)	70 MS/CIS 17 HC	7 CIS 36 RRMS 26 SPMS	MS/CIS: 42.8 HC: 41.6	25:62	Not reported	CIS 0.86 (0.85) RRMS 2 (1.5) SPMS 5.39 (1.34)	Mean % time on DMT CIS 32.5% RRMS 77.6% SPMS 52.8%	United States	LONG (4 years)	1.5T T1-weighted, T2-weighted, FLAIR, PD 0.9 × 0.9 × 5 mm voxels	Whole brain atrophy and GM atrophy were accelerated in more advanced stages of MS. Increasing GM atrophy accounted for whole brain atrophy entirely as rates of WM atrophy were constant in all MS groups. There were no differences in atrophy rates between HC and the CIS group who did not convert to MS during the 4 years. Annual rate of change in volume of WM, GM and whole brain atrophy within all MS groups was not correlated with age but was positively correlated with disease severity. After adjusting for age, rate of GM atrophy in the RRMS and SPMS groups was significantly higher than HCs atrophy rate. This indicates that GM volume loss caused by MS accelerates over time.
Ghione et al. (27)	1982 MS 351 HC	1,530 RRMS 371 SPMS 81 PPMS	MS: 46.8 HC: 44.8	1718:615	90.3% Caucasian 8.9% African American 0.8% Other	Not reported	15.7% were not on DMTs at first MRI	Not stated	LONG (6 mons to 10 years) *retrospective*	1.5T and 3T 3D T1-weighted, FLAIR 1 mm^3^ resolution	Development of brain atrophy manifests progressively in MS group, and occurs in a different pattern than that of HC aging. Percent lateral ventricular volume change (PLVVC) was increased across age in HCs as compared to MS group. Percent brain volume change (PBVC) decreased across age in both HC and MS groups.
Høgestøl et al. (28)	76 MS 3,443 HC (235 MRI data)	73 RRMS 1 SPMS 2 PPMS	MS: 34.8 HC: 47	1,028:2491	Not reported	Baseline median EDSS 2.0 (range 0–6)	22% were not on DMTs at first MRI	Norway	XS (HC) LONG (MS; 4.4 years)	1.5T 3D T1-weighted, T2-weighted, FLAIR; 3T 3D T1-weighted 1.2 × 1.25 × 1.25 mm voxels	On average, the MS group had a significantly higher brain age gap (biological brain age is older than chronological brain age) compared to HCs. In the MS group, compared to HCs, there was a high global brain age gap (4.4 years) and an even higher brain age gap (6.2 years) for subcortical/cerebellar brain regions. There was also an annual increase in brain age gap in the MS group. Progressive brain aging in the MS group was related with brain atrophy and WM lesion load (WMLL). Brain age gap and WMLL as well as brain age gap and global brain atrophy were shown to be positively correlated.
Jakimovski et al. (29)	2,199 MS/CIS	192 CIS 1,554 RRMS 453 SPMS/PPMS	46	548:1651	90.5% Caucasian 8.7% African American 0.8% Other	2.5 (IQR 1.5–4.5)	17.8% were not on DMTs	United States	XS	1.5T and 3T 3D T1-weighted, FLAIR 1 mm^3^ resolution	Compared to age-matched females with MS, a greater proportion of males with MS were diagnosed with progressive MS and had lower whole brain volume, lower GM volume and increased lateral ventricular volume. These findings remained significant after correcting for head size, MS groups and treatment. The sex differences were not evident in individuals who were 60+ years old.
Jakimovski et al. (30)	112 MS 184 HC	59 RRMS 48 SPMS 5 PPMS	MS: 60.3 HC: 59.5	79:217	Not reported	3.5 (IQR 2.3–6)	Not reported	Not stated	XS	3T 3D T1-weighted, T2-weighted, FLAIR 1 × 1 × 3 mm voxels	The MS group had lower volumes compared to age-matched HCs in whole brain, WM, GM, deep GM, thalamus, caudate, putamen, globus pallidus and hippocampus, lateral ventricular regions.
Kassubek et al. (31)	33 MS 60 HC	33 RRMS	MS: 34.9 HC: 50.8	35:58	Not reported	Mean 2.4 (range 0–6.5)	Not reported	Germany	XS	1.5T T1-weighted, T2-weighted 1 mm^3^ resolution	The MS group had significantly higher whole brain atrophy compared to age-matched HCs. Brain atrophy did also increase with age in HCs. In the MS group, whole brain atrophy was significantly increased in correlation with longer disease duration and higher disability.
Krysko et al. (32)	516 MS/CIS	RRMS 367 CIS 80 SPMS 47 PPMS 17 PRMS 4 Unclear 1	42.6	160:356	Not reported	Median 1.5 (range 0–7)	29.3% were not on DMTs	United States	Observational LONG (5, 10 years)	3T 3D T1-weighted, T2-weighted, PD Resolution not reported	Shorter telomere length was shown to be associated with disability and brain atrophy (total brain volume) in the MS group, independent of chronological age and disease duration. The authors conclude that biological aging contributes to brain degeneration in pwMS and that individual variability in biological aging may contribute to heterogeneity in MS course.
Kuusisto et al. (33)	19 MS 19 HC	8 RRMS 8 SPMS 3 PPMS	51.6	12:26	Not reported	Monozygotic twins median 5.0 (range 2–7) Dizygotic twins median 2.5 (range 1–8)	2 monozygotic twins were on DMTs 5 dizygotic twins were on DMTs	Finland	XS *twin matched*	1.5T 3D T1-weighted, T2-weighted, FLAIR Resolution not reported	There were no significant differences found in brain and spinal cord volumes between twins in the MS group and their co-twins in the HC group. Results support that brain volume in twins is highly heritable. All twins in MS group had focal brain WM lesions and 3 had spinal cord lesions and 14 out of 19 fulfilled Barkhof MRI criteria. 9 out of 19 co-twins in the HC group had focal brain WM lesions but all lesions were significantly smaller than in the twins from the MS group and none fulfilled the Barkhof MRI criteria. There was no evaluation of associations between WM lesions and total brain atropy.
Martola et al. (34)	37 MS	16 RRMS 17 SPMS 4 PPMS	42	11:26	Not reported	Specific scores not reported, although EDSS was examined in relation to brain metrics	64.86% on Interferon treatment 35.14% Nontreatment	Sweden	LONG (7–10 years)	1.5T T1-weighted 0.9 × 1.15 × 5 mm	There were evident linear annual decreases in brain volume, annual increases in left and right lateral ventricle volumes and linear annual increases in third ventricle volumes over the 4 decades of disease duration represented. There were no indications that differences between left and right ventricles would increase/decrease over disease duration. There was also no evidence of a specific timepoint altering brain atrophy or of acceleration or decline of atrophy over time. Corpus callosum atrophy and total brain atrophy were shown to be positively correlated with each other. There were no differences in atrophy patterns and no dependence on corpus callosum or ventricular size differences between MS courses. Disability increased with increases in atrophy rates of third and lateral ventricles. Older individuals showed larger ventricles at entry but rates of atrophy progression did not significantly differ from younger individuals.
Newbould et al. (35)	38 MS 11 HC	36 RRMS 2 SPMS	“Young” MS”: 30.4 “Older” MS: 49.7 HC: 48.5	11:38	Not reported	Young 2.5 (range 2–6) Older 3.8 (range 2–6)	Not reported	United Kingdom	XS	3T 3D T1-weighted, FLAIR, MT 1 mm^3^ resolution	Despite variable MS courses, brain atrophy seems to uniformly progress over longer periods of time. Normal aging of HCs from other studies in same age spans shows no signs of elevated age-related brain atrophy, but lack of HCs in this study limited the authors from determining how much MS disease added to annual brain atrophy rates caused by normal aging. Despite matching for disease duration and recording no significant WM lesion volume differences, there were strong magnetization transfer ratio (MTR) differences in WM lesions between the young and older MS groups. This implies that aging in MS exerts a direct negative effect on CNS myelin integrity in WM lesions that is reflected in MTR and also suggests that aging-related processes modify the tissue response to inflammatory injury and its clinical outcome correlates in MS.
Tiberio et al. (36)	21 MS 10 HC	21 RRMS	MS: 37.5 HC: 37.1	11:20	Not reported	Median 1 (Range 0–3)	7/21 participants started interferon B treatment in the first year	United Kingdom	LONG (2 years)	1.5T T1-weighted, T2-weighted, PD 1.2 × 1.2 × 1.5 mm voxels	A decrease in GM volume over 2 years was seen in the MS group, compared to HCs. There was no change observed in WM volume. However, WM volume change was seen with the MS group who had gadolinium-enhancing lesion loads. The authors concluded that GM atrophy but not WM atrophy was observed early in the clinical course of RRMS. Fluctuations in WM lesions are related to volume changes in WM over two years.
Tortorella et al. (37)	200 MS	172 RRMS 28 SPMS	35.3	81:119	Not reported	Median 2 (range 1–5.5)	No participants were on DMTs	Italy	XS *retrospective*	1.5T T1-weighted, T2-weighted 1.3 × 1 × 5 mm voxels	Frequency and number of gadolinium-enhancing lesions was higher in younger, less disabled individuals with MS with greater disease activity in the 2 years before MRI examination. Main changes in enhancement risk occurs after 35 years of age. In a previous study, they also had found a faster age-related rate of progression after a similar age. This supports that lesion burden and new inflammatory lesion formation in articulate parts of CNS are main pathogenetic mechanisms of disability in younger individuals with MS whereas neurodegenerative mechanisms might contribute to neuronal decline in older, higher disabled individuals with MS.

Includes, sample size, age, sex, geographic location, methods and results; XS, cross-sectional; LONG, longitudinal.

*Note that this study overlaps with Azevedo (17).

### Study quality analysis

The AXIS tool (38) was used to assess study quality in both cross-sectional and longitudinal studies included in this review. Quality was assessed by 20 questions that allow for evaluation of any pitfalls that studies may have. Table 2 shows the breakdown of quality analysis across each of the 20 questions for the 21 included studies.

**Table 2:** AXIS tool responses to assess qualitative features of papers that were used in this review.

	Clear aims/objectives?	Appropriate study design?	Sample size justified?	Target population defined?	Sample taken from appropriate population?	Appropriate sample selection process?	Addressed/categorized non-responders?	Appropriate risk factor/outcomes?	Appropriate risk factor/outcome measures?	Appropriate statistical significance methods?	Methods sufficiently described?	Basic data adequately described?	Non-response bias?	Non-responder information described?	Results internally consistent?	Results described for all analyses?	Discussions/conclusions justified by results?	Limitations discussed?	Funding sources/conflicts of interest?	Ethical approval/consent attained?
Azevedo et al. (17)	Y	Y	U	Y	U	U	N/A	Y	Y	Y	Y	Y	U	U	Y	Y	Y	Y	U	Y
Azevedo et al. (18)	Y	Y	Y	Y	—	—	Y	Y	Y	Y	Y	Y	U	—	Y	Y	Y	Y	—	Y
Baird et al. (19)	Y	Y	Y	Y	Y	Y	U	Y	Y	Y	U	Y	—	—	Y	Y	Y	Y	—	Y
Bishop et al. (20)	Y	Y	U	Y	—	U	U	Y	Y	Y	Y	Y	U	—	Y	Y	Y	Y	—	Y
Bove et al. (21)	Y	Y	Y	Y	Y	Y	N/A	Y	Y	Y	Y	Y	—	—	Y	Y	Y	Y	—	Y
Cole et al. (22)	Y	Y	Y	Y	Y	Y	N/A	Y	Y	Y	Y	U	—	N/A	U	Y	Y	Y	—	Y
Erramuzpe et al. (23)	Y	Y	Y	Y	Y	U	U	Y	Y	U	U	Y	U	U	Y	Y	Y	Y	—	U
Eshaghi et al. (24)	Y	Y	Y	Y	—	U	N/A	Y	Y	Y	Y	Y	—	N/A	U	Y	Y	Y	—	Y
Eshaghi et al. (25)	Y	Y	U	Y	Y	Y	N/A	Y	Y	Y	Y	Y	N/A	N/A	Y	Y	Y	Y	U	Y
Fisher et al. (26)	Y	Y	—	Y	U	U	—	Y	Y	Y	Y	Y	U	Y	Y	Y	Y	Y	U	Y
Ghione et al. (27)	Y	Y	U	Y	Y	Y	Y	Y	Y	Y	U	Y	—	U	Y	Y	Y	Y	—	Y
Høgestøl et al. (28)	Y	Y	Y	Y	Y	Y	—	Y	Y	Y	Y	Y	U	—	Y	Y	Y	Y	U	Y
Jakimovski et al. (29)	Y	Y	Y	Y	Y	Y	N/A	Y	Y	Y	Y	Y	—	N/A	U	Y	Y	Y	—	Y
Jakimovski et al. (30)	Y	Y	Y	Y	Y	Y	U	Y	Y	Y	Y	Y	U	N/A	Y	Y	Y	Y	—	Y
Kassubek et al. (31)	Y	Y	Y	Y	Y	Y	N/A	Y	Y	Y	Y	Y	N/A	N/A	U	Y	Y	—	—	Y
Krysko et al. (32)	Y	Y	Y	Y	Y	Y	Y	Y	Y	Y	Y	U	—	—	U	Y	Y	Y	—	Y
Kuusisto et al. (33)	Y	Y	Y	Y	Y	Y	U	Y	Y	Y	Y	Y	U	U	Y	Y	Y	Y	—	Y
Martola et al. (34)	Y	Y	Y	Y	Y	Y	Y	Y	Y	Y	Y	Y	—	—	Y	Y	Y	Y	—	Y
Newbould et al. (35)	Y	Y	U	Y	U	U	U	Y	Y	Y	U	Y	U	—	Y	Y	Y	Y	U	Y
Tiberio et al. (36)	Y	Y	Y	Y	—	Y	N/A	Y	Y	—	Y	Y	—	—	U	Y	Y	Y	—	Y
Tortorella et al. (37)	Y	Y	Y	Y	Y	Y	N/A	Y	Y	Y	Y	Y	N/A	N/A	Y	Y	Y	—	U	Y

Y, yes; N, no; U, unclear; N/A, not applicable.

### Main findings

Overall, 21 studies were included in the final analyses. A description of each study, including sample size, MS group breakdown, mean age and sex of participants, study geography, study type and duration, type of scans acquired/analyzed, and a summary of study results, are included in Table 1. The findings are discussed further in the context of the research questions guiding this review in the discussion section below.

## Discussion

MS is a chronic neurological disorder that is characterized by lesions in the CNS and typically diagnosed in young to mid-adulthood. Relatively less research has focused on older adults with MS, although recent questions have emerged about whether there is an accelerated aging effect for pwMS. The current study involved a systematic review of the literature that used structural MRI methods to examine changes in the brain specific to aging with MS. This review considered cross sectional studies that examined differences between pwMS and healthy controls, as well as longitudinal studies tracking changes in the brain in pwMS over time with an aim to answer several specific questions. Herein, we pose each of the pre-determined questions along with conditional answers based on the extant literature.

### How does the brain differ or change with age in pwMS?

With an aim to answer this overarching question, the search terms of the current review included both gray and white matter as well as specific imaging techniques, such as diffusion tensor imaging (DTI), which provides metrics of white matter microstructure. However, there were no studies that focused on white matter using DTI and although several studies examined total white matter volume, the vast majority of studies focused on gray matter volume using high resolution T1-weighted anatomical MRI scans.

Of the 21 identified studies, 13 compared pwMS and healthy controls and all of these revealed significant structural brain differences. Notably, some studies focused on broad metrics such as whole brain volume, while other studies extracted values representing gray and white matter volume or white matter lesion load. Several studies also extracted volumes for particular regions or carried out voxel-based morphometry analyses. Although there was variability in the regions examined across studies, 4 studies reported decreased whole brain volume in pwMS (27, 29–31, 39), 6 reported decreased gray matter volume (18, 24, 25, 29, 30, 36, 39), 6 reported decreased volume in subcortical gray matter structures (17, 18, 20, 24, 25, 29, 30) (particularly in the thalamus and basal ganglia) and 3 reported decreases in white matter based on volume or lesion load (19, 29, 30, 33). Notably, 6 of these studies examined how atrophy rates in healthy controls compared to pwMS. These studies suggested that pwMS showed accelerated rates of atrophy in the thalamus (17, 18) and other gray matter regions (24, 25, 39).

Three studies investigated predicted “brain age” using various algorithms and found that pwMS had significantly larger predicted brain age differences (higher biological age compared to chronological age) in comparison to healthy controls which also suggests accelerated aging in pwMS (22, 23, 28). Finally, a unique study from Krysko et al. (32) established that short leukocyte telomere length was associated with disability and brain atrophy (measured as total brain volume) in pwMS. The authors suggest that based on this finding, biological aging may contribute to disability worsening in MS (32).

Taken together, the studies included in this review provide evidence that supports acceleration of whole brain and gray matter atrophy as pwMS age when compared to normal aging in people without MS. Gray matter and subcortical regions including the thalamus appear particularly vulnerable to faster atrophy with age in pwMS as compared to healthy normal aging controls, although there were limited numbers of studies that focused on specific regions and on white matter which limits the conclusions that can be drawn from the literature.

### Are there specific MRI findings in older pwMS compared to younger pwMS?

Of the 21 studies reviewed, 6 used MRI to examine brain structure differences in younger and older pwMS using a mix of cross-sectional and longitudinal approaches (18, 20, 21, 34, 35, 37). Using a cross-sectional approach, Newbould et al. (35) compared younger (30.4 years) and older (49.7 years) pwMS and found strong magnetization transfer ratio (MTR) differences between the two age groups within white matter lesions. This suggests decreased myelin integrity in the older age group. Bishop et al. (20) also took a cross-sectional approach and compared both younger (30.4 years) and older (48.7 years) pwMS to age-matched healthy controls. They found similar patterns of atrophy for each age group with MS in the putamen, thalamus and nucleus accumbens, but significantly more atrophy in the hippocampus and caudate in the younger group. A retrospective, cross-sectional approach was used by Tortorella et al. (37) to examine gadolinium-enhancing lesions in MS populations. They found that enhanced lesions were more frequently found in younger patients with lower disability. They also showed that the change in risk for gadolinium-enhancing lesions occurs at 35 years of age in pwMS. The authors proposed that these lesions may contribute to disease mechanisms in younger pwMS, whereas there are likely age-related neurodegenerative mechanisms occurring in older pwMS. Bove et al. (21) used a mixed design with a longitudinal component to examine changes in slopes of MRI extracted metrics including total brain volume and white matter lesion load over 2 years in pwMS who were under (younger group) or over (older group) 50 years of age (42 years and 58 years, respectively). The authors found no significant differences in slope of change as a function of age for either metric. Martola et al. (34) also used longitudinal observations to examine changes in brain volume at three time points over a decade in pwMS (ranging in age from 24–65 years) and found uniform progression of atrophy over time. Lastly, an interesting study by Azevedo et al. (18) examined annual MRI scans from 520 participants with RRMS over 5 years and found that the contributions of normal aging and MS-related atrophy differed by brain region. Findings revealed that the contributions of aging became greater with each decade from 30 to 60 years of age, while the contribution of MS-related atrophy lessened. In contrast, the contributions of aging and MS-related atrophy were relatively more stable over time in the putamen and caudate. These findings underscore the need for more comprehensive regional analyses to better understand patterns of brain atrophy in aging pwMS.

The various approaches to study design and analyses create challenges and limitations in synthesizing the results to date. Although there is some evidence that aging may have differential effects on brain atrophy in pwMS across the lifespan as compared to normal aging, more research is needed. In particular, the use of regional approaches in older age groups (e.g., 60+ years) that have not been well captured require further examination.

### Are there structural differences in the brain as a function of sex in aging pwMS?

Typically, MS affects 3 times more females than males, although disability progression occurs quicker in males (40). Of the 21 studies included, 2 examined aging as a function of sex in pwMS (21, 29, 30). Although Bove et al. (21) did not find differences in rate of whole brain atrophy as a function of age, their findings did reveal significantly greater disease severity (based on EDSS scores) and whole brain atrophy in males compared to females. Further, Jakimovski et al. (29, 30) highlighted similar findings with males demonstrating lower whole brain and gray matter volumes as well as increased ventricular volume compared to females. However, this paper also showed that after the age of 60, there were no evident differences in brain structure between males and females with progressive MS (29, 30).

As evidenced by our literature search, there are a limited number of studies examining sex differences in aging pwMS. However, the evidence to date suggests that males have greater brain atrophy than females with age. One potential factor that may be attributed to the differences seen between sexes is menopause as it has been suggested to affect brain atrophy patterns in aging females with MS (41). Another factor may relate to the course of MS in males, as males have been shown to have more rapid disability accumulation and cognitive decline than females (42). More research including longitudinal studies that capture changes from age 40 to 60 in both females and males are essential to understanding the potential effects of sex on age-related atrophy over time and whether such differences are also stable over time.

### Conclusion, limitations, and future directions

The current review represents the first synthesis of the literature examining brain aging in pwMS. It was hypothesized that there would be age-related differences in brain structure that were specific to pwMS and beyond the changes associated with typical aging, with older adults showing greater atrophy. Indeed, the literature revealed that cortical and subcortical gray matter are vulnerable to excess atrophy with age in pwMS. Predicted brain age was also consistently higher and atrophy rates were accelerated in pwMS compared to healthy controls. There were also indications that certain brain regions may become more prone to atrophy as pwMS age and that males may experience greater atrophy than females. However, more research is imperative to fully address each of the research questions posed in this review and there are several strengths and limitations within the current review, as well as within the literature that should be addressed.

With regards to the current review, strengths included the broad approach taken to search terms in combination with the focused questions on age related changes as measured by MRI in pwMS. The regional patterns and characteristics that influence brain atrophy for pwMS have important implications for clinical practice. In particular, understanding expected changes related to aging and whether there is accelerated neurodegeneration in MS can assist with interpretation of MRI findings for older adults with MS.

Conversely, a limitation was that studies examining correlations between structural brain changes with age and clinical variables were beyond the scope of the current review. Several studies that met the criteria for review happened to include comparisons of brain atrophy patterns and clinical outcomes (e.g., disease severity) with significant findings. Specifically examining such relationships between age related atrophy and symptoms of MS could form the topic of a separate focused systematic review.

Within the literature, the quality of the reviewed studies was consistently high. Assessment of study quality with the AXIS tool revealed that all of the studies in the current review clearly stated their objectives and designed their study appropriately, using proper outcome variables. The majority of studies were also clear on how significance was determined, and the conclusions made within each study were consistent with the presented results. The main limitations of the literature became apparent when synthesizing the findings. Specifically, there was heterogeneity in methods, including widely variable sample sizes (ranging from 19 to 2,199 pwMS), acquisition techniques (e.g., field strength, imaging sequences, parameters), analysis approaches (e.g., whole brain, gray matter volume, white matter lesion load, voxel-based morphometry, various brain age algorithms), and study designs (e.g., different age groups, study protocols) that yielded findings that are difficult to weigh and integrate across studies. As mentioned previously, there was a distinct lack of studies that examined aging pwMS using diffusion tensor imaging derived metrics of white matter microstructure. Given that changes in myelination and axonal integrity are characteristic of MS and that DTI is sensitive to both lesions and changes in normal appearing white matter, this technique could provide important information on the aging brain for pwMS. Additionally, in most of the included studies, the majority of participants had RRMS, although SPMS most commonly develops over the disease course of people with RRMS and should be better represented in studies on aging. Relatedly, people with different subtypes of MS may be more or less likely to be on disease modifying treatments (DMTs). Many, but not all, of the studies reviewed reported on DMTs, which was higher in people with RRMS than other subtypes. The differences and changes in brain volume detected in these studies were largely present in the context of DMT use. Although it is beyond the scope of the current review to determine whether DMT use has a modifying effect on neurodegeneration, ongoing research should report DMT use so that future reviews can examine whether any accelerated aging effects are muted by treatment (as current cohorts on these treatments age).

Importantly, previous research has shown that African Americans exhibit more rapid neurodegeneration than Caucasian Americans (43); however, most studies did not report on race or ethnicity and those that did report these characteristics had a majority of white participants. Moving forward, it will be important for research to be inclusive and involve transparent reporting.

In terms of other directions for future research, there is a clear need for large scale studies that use multimodal MRI acquisition methods to capture changes in both males and females over time, particularly in middle to older adulthood. Such studies would essentially aid the understanding of how the brain ages in pwMS, which will have implications for both the conceptualization of the disease course of MS and the interpretation of intervention related changes in the brain as pwMS age.

## Author contributions

NT and IT drafted the initial manuscript. All authors were involved in conceptualizing the review, reviewing the literature, and critical revisions.

## Funding

This project was supported by the endMS Scholar Program for Researchers IN Training (SPRINT) program by the Multiple Sclerosis Society of Canada.

## Conflict of interest

The authors declare that the research was conducted in the absence of any commercial or financial relationships that could be construed as a potential conflict of interest.

## Publisher’s note

All claims expressed in this article are solely those of the authors and do not necessarily represent those of their affiliated organizations, or those of the publisher, the editors and the reviewers. Any product that may be evaluated in this article, or claim that may be made by its manufacturer, is not guaranteed or endorsed by the publisher.
