# Supplementary material for: How does the brain age in individuals with multiple sclerosis? A systematic review
Source: Front Neurol. 2023 Jun 30;14:1207626. doi: 10.3389/fneur.2023.1207626 (PMC10349663; doi:10.3389/fneur.2023.1207626)
Supplement: Supplementary file 1 [file Data_Sheet_1.docx]

Systematic Review Search Terms

Search was run on September 22, 2021

**SEARCH TERMS: PUBMED**

| Search: **((multiple sclerosis) AND (MRI)) AND (aging)** search 1  Filters: **Humans, English, Adult: 19+ years** Sort by: **Most Recent**  (("multiple sclerosis"[MeSH Terms] OR ("multiple"[All Fields] AND "sclerosis"[All Fields]) OR "multiple sclerosis"[All Fields]) AND ("magnetic resonance imaging"[MeSH Terms] OR ("magnetic"[All Fields] AND "resonance"[All Fields] AND "imaging"[All Fields]) OR "magnetic resonance imaging"[All Fields] OR "mri"[All Fields]) AND ("aging"[MeSH Terms] OR "aging"[All Fields] OR "ageing"[All Fields])) AND ((humans[Filter]) AND (english[Filter]) AND (alladult[Filter]))  **Translations**  **multiple sclerosis:** "multiple sclerosis"[MeSH Terms] OR ("multiple"[All Fields] AND "sclerosis"[All Fields]) OR "multiple sclerosis"[All Fields]  **MRI:** "magnetic resonance imaging"[MeSH Terms] OR ("magnetic"[All Fields] AND "resonance"[All Fields] AND "imaging"[All Fields]) OR "magnetic resonance imaging"[All Fields] OR "mri"[All Fields]  **aging:** "aging"[MeSH Terms] OR "aging"[All Fields] OR "ageing"[All Fields]  Search: **(((multiple sclerosis) AND (grey matter)) AND (imaging)) AND (aging)** search 2  Filters: **Humans, English, Adult: 19+ years** Sort by: **Most Recent**  (("multiple sclerosis"[MeSH Terms] OR ("multiple"[All Fields] AND "sclerosis"[All Fields]) OR "multiple sclerosis"[All Fields]) AND ("gray matter"[MeSH Terms] OR ("gray"[All Fields] AND "matter"[All Fields]) OR "gray matter"[All Fields] OR ("grey"[All Fields] AND "matter"[All Fields]) OR "grey matter"[All Fields]) AND ("image"[All Fields] OR "image s"[All Fields] OR "imaged"[All Fields] OR "imager"[All Fields] OR "imager s"[All Fields] OR "imagers"[All Fields] OR "images"[All Fields] OR "imaging"[All Fields] OR "imaging s"[All Fields] OR "imagings"[All Fields]) AND ("aging"[MeSH Terms] OR "aging"[All Fields] OR "ageing"[All Fields])) AND ((humans[Filter]) AND (english[Filter]) AND (alladult[Filter]))  **Translations**  **multiple sclerosis:** "multiple sclerosis"[MeSH Terms] OR ("multiple"[All Fields] AND "sclerosis"[All Fields]) OR "multiple sclerosis"[All Fields]  **grey matter:** "gray matter"[MeSH Terms] OR ("gray"[All Fields] AND "matter"[All Fields]) OR "gray matter"[All Fields] OR ("grey"[All Fields] AND "matter"[All Fields]) OR "grey matter"[All Fields]  **imaging:** "image"[All Fields] OR "image's"[All Fields] OR "imaged"[All Fields] OR "imager"[All Fields] OR "imager's"[All Fields] OR "imagers"[All Fields] OR "images"[All Fields] OR "imaging"[All Fields] OR "imaging's"[All Fields] OR "imagings"[All Fields]  **aging:** "aging"[MeSH Terms] OR "aging"[All Fields] OR "ageing"[All Fields]  Search: **(((multiple sclerosis) AND (white matter)) AND (imaging)) AND (aging) search 3**  Filters: **Humans, English, Adult: 19+ years** Sort by: **Most Recent**  (("multiple sclerosis"[MeSH Terms] OR ("multiple"[All Fields] AND "sclerosis"[All Fields]) OR "multiple sclerosis"[All Fields]) AND ("white matter"[MeSH Terms] OR ("white"[All Fields] AND "matter"[All Fields]) OR "white matter"[All Fields]) AND ("image"[All Fields] OR "image s"[All Fields] OR "imaged"[All Fields] OR "imager"[All Fields] OR "imager s"[All Fields] OR "imagers"[All Fields] OR "images"[All Fields] OR "imaging"[All Fields] OR "imaging s"[All Fields] OR "imagings"[All Fields]) AND ("aging"[MeSH Terms] OR "aging"[All Fields] OR "ageing"[All Fields])) AND ((humans[Filter]) AND (english[Filter]) AND (alladult[Filter]))  **Translations**  **multiple sclerosis:** "multiple sclerosis"[MeSH Terms] OR ("multiple"[All Fields] AND "sclerosis"[All Fields]) OR "multiple sclerosis"[All Fields]  **white matter:** "white matter"[MeSH Terms] OR ("white"[All Fields] AND "matter"[All Fields]) OR "white matter"[All Fields]  **imaging:** "image"[All Fields] OR "image's"[All Fields] OR "imaged"[All Fields] OR "imager"[All Fields] OR "imager's"[All Fields] OR "imagers"[All Fields] OR "images"[All Fields] OR "imaging"[All Fields] OR "imaging's"[All Fields] OR "imagings"[All Fields]  **aging:** "aging"[MeSH Terms] OR "aging"[All Fields] OR "ageing"[All Fields]  **OFFICIAL SEARCH TERM: PSYCINFO** |
| --- |

APA PsycInfo <1806 to September Week 2 2021>

1 ("multiple sclerosis" and "MRI" and "AGING").mp. [mp=title, abstract, heading word, table of contents, key concepts, original title, tests & measures, mesh] 25

2 limit 1 to (human and english language) 22

3 limit 2 to adulthood <18+ years> 10

1 ("multiple sclerosis" and "grey matter" and "imaging" and "aging").mp. [mp=title, abstract, heading word, table of contents, key concepts, original title, tests & measures, mesh] 2

2 limit 1 to (human and english language) 2

3 limit 2 to adulthood <18+ years> 1

1 ("multiple sclerosis" and "gray matter" and "imaging" and "aging").mp. [mp=title, abstract, heading word, table of contents, key concepts, original title, tests & measures, mesh] 7

2 limit 1 to (human and english language) 7

3 limit 2 to adulthood <18+ years> 5

1 ("multiple sclerosis" and "white matter" and "imaging" and "aging").mp. [mp=title, abstract, heading word, table of contents, key concepts, original title, tests & measures, mesh] 9

2 limit 1 to (human and english language) 8

3 limit 2 to adulthood <18+ years> 1
